# Supplementary material for: Development of a polygenic risk score to improve screening for fracture risk: A genetic risk prediction study
Source: PLoS Med. 2020 Jul 2;17(7):e1003152. doi: 10.1371/journal.pmed.1003152 (PMC7331983; doi:10.1371/journal.pmed.1003152)
Supplement: S1 Checklist — (DOCX) [file pmed.1003152.s001.docx]

## GRIPS Checklist

Reporting recommendations for evaluations of risk prediction models that include genetic variants:

| **Report section** | **Item No** | **Recommendation** | **Done in this manuscript (if not, why)?** |
| --- | --- | --- | --- |
| **Title and abstract** | |  |  |
|  | 1 | (*a*) Identify the article as a study of risk prediction using genetic factors | **Yes** |
|  |  | *(b*) Use recommended keywords in the abstract: genetic or genomic, risk, prediction | **Yes** |
|  |  |  |  |
| **Introduction** | | |  |
| Background and rationale | 2 | Explain the scientific background and rationale for the prediction study | **Yes** |
| Objectives | 3 | Specify the study objectives and state the specific model(s) that is/are investigated. State if the study concerns the development of the model(s), a validation effort, or both | **Yes** |
|  |  |  |  |
| **Methods** | | |  |
| Study design and setting | 4* | Specify the key elements of the study design and describe the setting, locations, and relevant dates, including periods of recruitment, follow-up, and data collection | **Not applicable, as no recruitment was performed. All data was collected as part of existing studies that are cited.** |
| Participants | 5* | Describe eligibility criteria for participants, and sources and methods of selection of participants | **Yes** |
| Variables: definition | 6* | Clearly define all participant characteristics, risk factors and outcomes. Clearly define genetic variants using a widely used nomenclature system | **Yes** |
| Variables: assessment | 7* | (*a*) Describe sources of data and details of methods of assessment (measurement) for each variable | **Yes** |
|  |  | (*b*) Give a detailed description of genotyping and other laboratory methods | **Not applicable, as genotype data is collected from existing studies that are cited.** |
| Variables: coding | 8 | (*a*) Describe how genetic variants were handled in the analyses | **Yes** |
|  |  | (*b*) Explain how other quantitative variables were handled in the analyses. If applicable, describe which groupings were chosen, and why | **Yes** |
| Analysis: risk model construction | 9 | Specify the procedure and data used for the derivation of the risk model. Specify which candidate variables were initially examined or considered for inclusion in models. Include details of any variable selection procedures and other model building issues. Specify the horizon of risk prediction (eg, 5 year risk) | **Yes** |
| Analysis: validation | 10 | Specify the procedure and data used for the validation of the risk model | **Yes** |
| Analysis: missing data | 11 | Specify how missing data were handled | **Yes** |
| Analysis: statistical methods | 12 | Specify all measures used for the evaluation of the risk model including, but not limited to, measures of model fit and predictive ability | **Yes** |
| Analysis: other | 13 | Describe all subgroups, interactions, and exploratory analyses that were examined | **Yes** |
| **Results** | | |  |
| Participants | 14* | Report the numbers of individuals at each stage of the study. Give reasons for non-participation at each stage. Report the number of participants not genotyped and reasons why they were not genotyped | **Yes** |
| Descriptives: population | 15* | Report demographic and clinical characteristics of the study population, including risk factors used in the risk modelling | **Yes** |
| Descriptives: model estimates | 16 | Report unadjusted associations between the variables in the risk model(s) and the outcome. Report adjusted estimates and their precision from the full risk model(s) for each variable | **Yes** |
| Risk distributions | 17* | Report distributions of predicted risks and/or risk scores | **Yes** |
| Assessment | 18 | Report measures of model fit and predictive ability, and any other performance measures, if pertinent. | **Yes** |
| Validation | 19 | Report any validation of the risk model(s) | **Yes** |
| Other analyses | 20 | Present results of any subgroup, interaction, or exploratory analyses, whenever pertinent | **Yes** |
|  |  |  |  |
| **Discussion** | | |  |
| Limitations | 21 | Discuss limitations and assumptions of the study, particularly those concerning study design, selection of participants, and measurements and analyses, and discuss their impact on the results of the study. | **Yes** |
| Interpretation | 22 | Give an overall interpretation of results considering objectives, limitations, multiplicity of analyses, results from similar studies, and other relevant evidence | **Yes** |
| Generalisability | 23 | Discuss the generalisability and, if pertinent, the healthcare relevance of the study results | **Yes** |
|  |  |  |  |
| **Other** | | |  |
| Supplementary information | 24 | State whether databases for the analysed data, risk models, and/or protocols are or will become publicly available and if so, how they can be accessed | **Yes, as part of UK Biobank** |
| Funding | 25 | Give the source of funding and the role of the funders for the present study. State whether there are any conflicts of interest | **Yes** |
|  |  |  |  |
| ** Marked items should be reported for every population in the study.* | | |  |
