# Supplementary figures and images for: Development of a polygenic risk score to improve screening for fracture risk: A genetic risk prediction study
Source: PLoS Med. 2020 Jul 2;17(7):e1003152. doi: 10.1371/journal.pmed.1003152 (PMC7331983; doi:10.1371/journal.pmed.1003152)

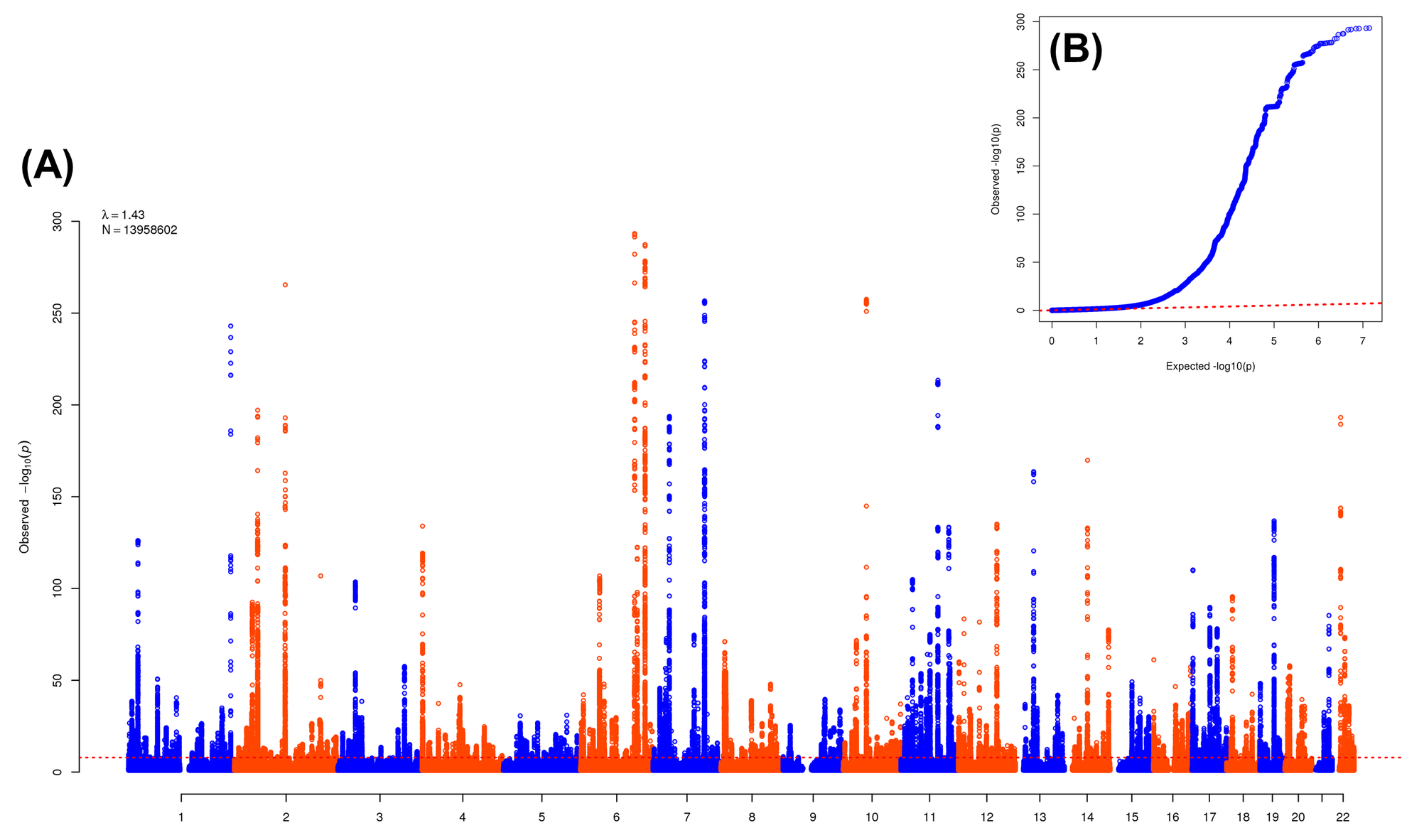

Supplement: S1 Fig — (A) Manhattan plot from GWAS of SOS. (B) QQ plot from GWAS of SOS. (TIFF) [file pmed.1003152.s002.tiff]

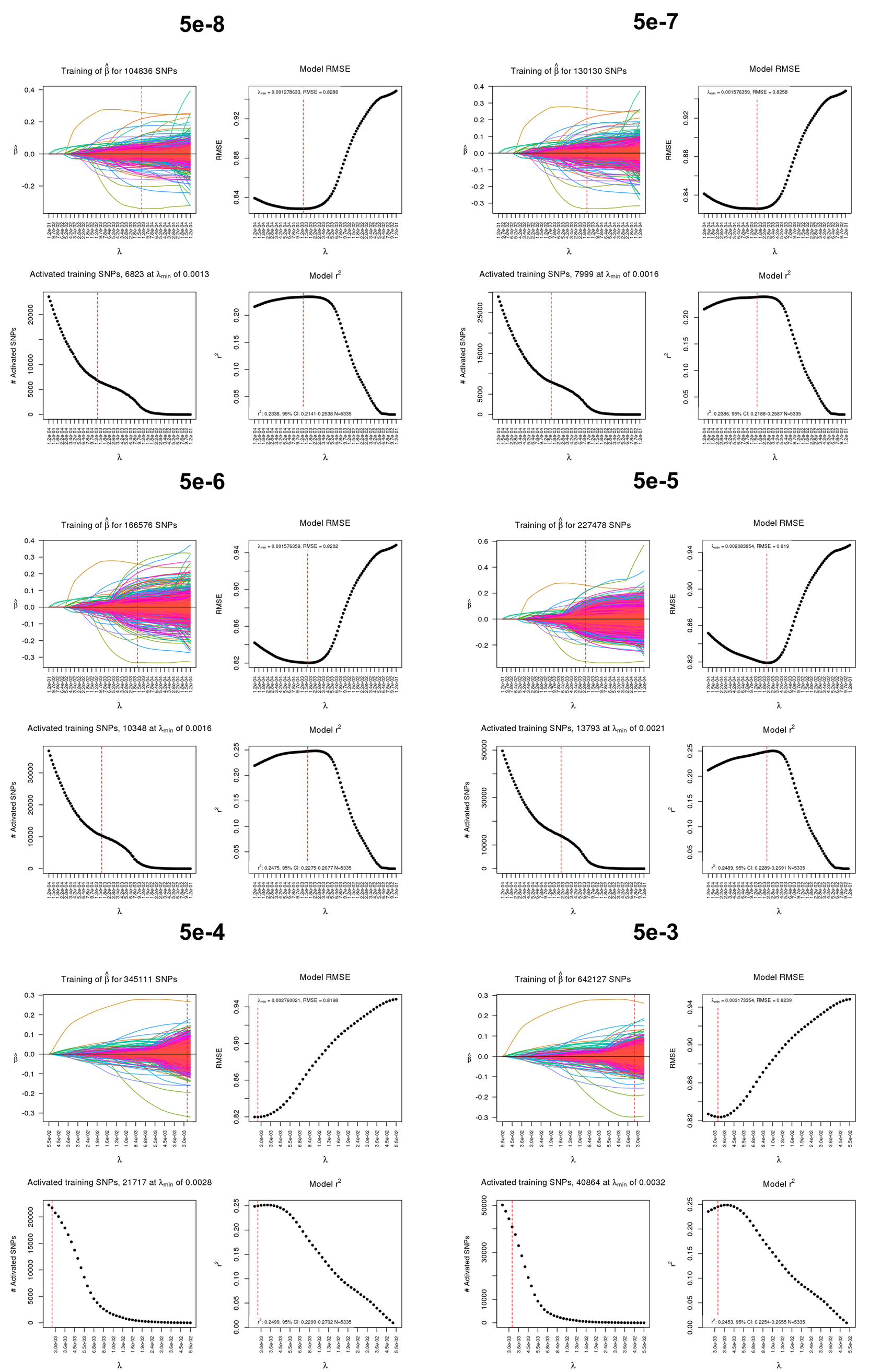

Supplement: S2 Fig — Each feature set consists of a set of SNPs associated with SOS at a specified p-value threshold (sub-panel titles). For each feature set, we fit a regularized model to the training set over a range of regularization constants (λ) (top left), with each λ resulting in a variable subset of activated features (bottom left). The model with the minimal root mean square error in the model selection set (top right) was selected to compare the variance explained (r2, bottom right among all feature sets. (TIFF) [file pmed.1003152.s003.tiff]

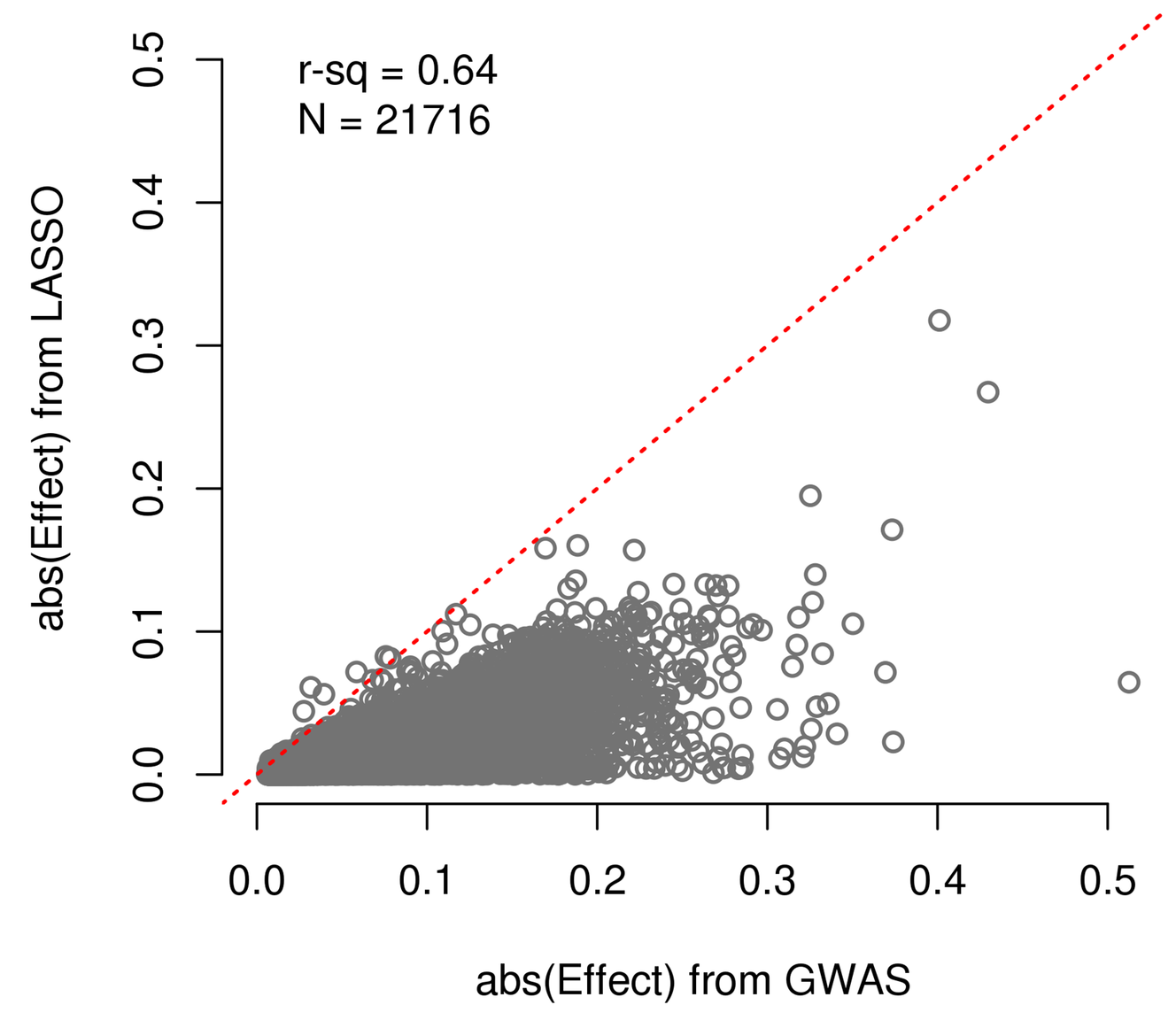

Supplement: S3 Fig — Activated SNPs are those SNPs chosen by the machine learning algorithm to be in gSOS, the final selected model. (TIFF) [file pmed.1003152.s004.tiff]

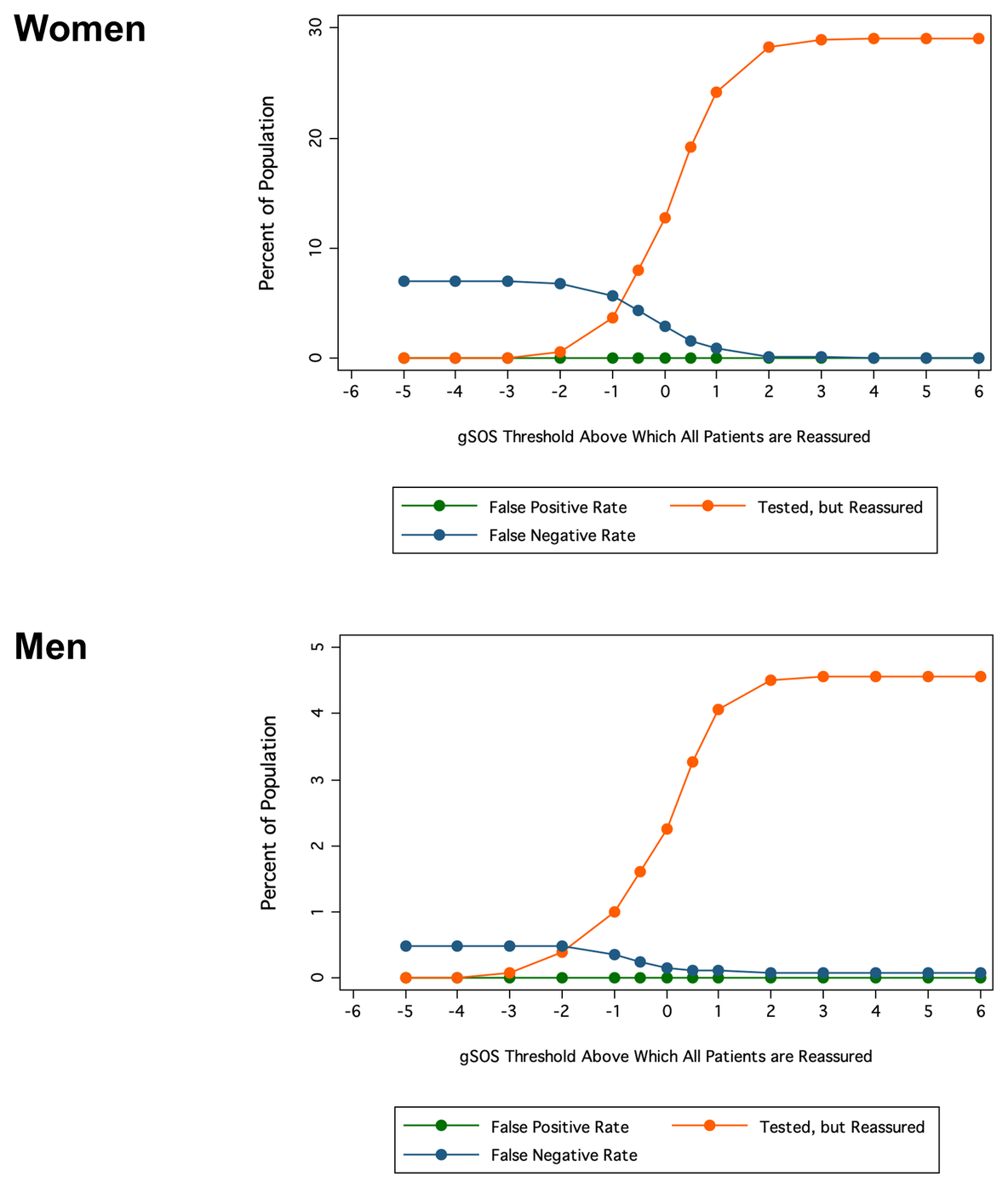

Supplement: S4 Fig — Results stratified by women (top) and men (bottom). (TIFF) [file pmed.1003152.s005.tiff]
