# Supplementary material for: Development of a polygenic risk score to improve screening for fracture risk: A genetic risk prediction study
Source: PLoS Med. 2020 Jul 2;17(7):e1003152. doi: 10.1371/journal.pmed.1003152 (PMC7331983; doi:10.1371/journal.pmed.1003152)
Supplement: S1 Text — (DOCX) [file pmed.1003152.s007.docx]

# Development of a polygenic risk score to improve screening for fracture risk: a genetic risk prediction study

# Supplemental Text

## Measurement of SOS and DXA-BMD

Full details of SOS measurement in UK Biobank are available here:

<https://biobank.ctsu.ox.ac.uk/crystal/docs/Ultrasoundbonedensitometry.pdf>

Full details of BMD measurement in UK Biobank are available here:

<http://biobank.ctsu.ox.ac.uk/crystal/docs/DXA_explan_doc.pdf>

### SOS measurement in UK Biobank

Briefly, a Sahara Clinical Bone Sonometer (Hologic Corporation, Bedford, Massachusetts, USA) was used for quantitative ultrasound assessment of calcanei in UK Biobank participants. Details of the complete protocol are publicly available on the UK Biobank website (see above URLs). Participants were initially measured at baseline (N = 487,428) and had their left calcaneus (N = 317,815), right calcaneus (N = 4,102) or both calcanei (N = 165,511) measured. Prior to quality control, ultrasound data were available for 488,366 individuals at either baseline and/or follow-up assessment. To reduce the impact of outlying measurements we first identified subjects that had both heels measured and removed those with highly discrepant (i.e. left vs. right) SOS and/or BUA measurements. To achieve this, subjects were stratified by sex and bivariate scatter plots comparing left and right heel measures of SOS and BUA were generated separately. Outliers were identified by manual inspection and removed. The same method was used to identify and remove individuals with highly discordant SOS v BUA measured for each heel. Strict quality control was thereafter applied to male and female subjects separately using the following exclusion thresholds: SOS [Male: (≤1,450 and ≥1,750 m/s), Female (≤1,455 and ≥1,700 m/s)] and BUA [Male: (≤27 and ≥138 dB/MHz), Female (≤22 and ≥138 dB/MHz)]. Individuals exceeding the threshold for SOS or BUA or both were removed from the analysis. A unique list of individuals with a valid measure for the left calcaneus (N = 477,380) and/or right (N = 181,953) were identified separately across the three time points. Individuals with a valid right calcaneus measure were included in the final data set when no left measures were available, giving a preliminary working dataset of N=481,100, (left = 475,724 and right = 5,376) unique individuals. Bivariate scatter plots of calcaneal measured were again visually inspected and 579 additional outliers were removed, leaving a total of 480,521 valid QUS measures (264,371 females and 216,150 males). Descriptive statistics of the cohort, after quality control, are detailed in Morris et al. [1].

### Femoral Neck BMD Measurement in UKBiobank (see Table S1 for other cohort details)

A GE-Lunar iDXA instrument was used to measure bone mineral density at the femoral neck in UK Biobank participants. Details of the complete protocol are publicly available on the UK Biobank website (“URLs”). We use data fields Data-Field 23299 and 23208, which correspond to left and right 'Femur neck BMD (bone mineral density)', respectively. There were 5,184 individuals with either left, right or both femoral neck BMD measurements, of which 4,834 are within the White British subset. Individuals who were not in the White British subset were excluded. To reduce the impact of outlying measurements in the White British subset, we excluded individuals who had left and right measurements that had greater than one standard deviation between left and right femoral neck BMD measurements (N=93). The remaining individuals (N=4,741) were assigned the left measurement if only left (N=1) or both (N=4739) were measured and assigned the right measurement if only right was measured (N=1). For all subsequent fracture prediction, femoral neck BMD was transformed to NHANES T-scores standardized to the mean of young women, calibrated to the GE-Lunar machine, using the formula provided by the manufacturer: NHANES T-Score = (femoral neck BMD [g/cm^2^]-1.03796) / 0.139.

## GWAS for SOS in UK Biobank

### Selection of SNPs and participants for GWAS

SNPs were first filtered for stringent quality control metrics, retaining only SNPs with a minor allele frequency (MAF) > 0.0005 and an imputation quality score (INFO score) >0.3 (**Fig 1**), leaving 13,958,791 SNPs for analysis. Ancestry was determined only for UK Biobank participants with high-quality genotype data (N=486,369). Using flashpca [2], genotype data comprising 38,539 LD-pruned HapMap3 SNPs (MAF > 0.01, minor allele count > 5, Hardy-Weinberg Equilibrium p-value < 1e^-6^) were projected onto previously computed principal components using the same SNPs set from 1000 Genomes Phase 3 (N=2,504). Cluster analysis as implemented in by the EMCluster R package was used to extract the UK Biobank individuals that are within the same cluster as the GBR (British in England and Scotland) 1000 Genomes population, resulting in 486,369 participants.

### Genome-wide association study (GWAS)

In the training dataset, tests of association were performed between SOS and each SNP, using an additive coding for the number of minor alleles, and with the BOLT-LMM software [3]. Age, sex, assessment centre, genotyping array and the first 20 principal components of ancestry were calculated from the white British subset and included as covariates in each of these models. These covariates were included to increase the power of the GWAS by reducing the residual error variance.

### Determining the White British Subset

To identify White British subset we used flashpca [2], on UK Biobank directly genotyped SNPs comprising 38,539 LD-pruned HapMap3 SNPs (MAF > 0.01, minor allele count > 5, Hardy-Weinberg Equilibrium p-value < 1e-6) which were projected onto previously computed principal components using the same SNPs set from 1000 Genomes Phase 3 (N=2,504). Cluster analysis as implemented in by the EMCluster R package was used to extract the UK Biobank individuals that are within the same cluster as the GBR (British in England and Scotland) 1000 Genomes population. For a full description of the method see [1]. The non-White British subset consists of the remaining individuals that were outside the cluster defined above.

## LASSO Regression and Polygenic Risk Scores Models to Predict SOS

### LASSO Regression model

For 6 p-value thresholds (**Table C in S1 Tables**), we selected all SNPs with p-values smaller than the threshold and used L1-penalized least absolute shrinkage and selection operator (LASSO) regression [4] to predict SOS in the training dataset. LASSO regression controls for model over-fitting by introducing a regularization term, λ, that shrinks all estimated parameters towards zero. This machine learning method achieves improved prediction when only a subset of all predictor variables independently contributes to the prediction. Importantly, this subset of predictors need not be genome-wide significant, nor need they be independent of each other. Only SNP data was used to build these LASSO models, and the number of SNPs considered ranged from 642,127 to 104,836. We used the biglasso implementation of LASSO given the size of the dataset [5]. The biglasso R package uses an out-of-memory matrix (implemented by the bigmemory R package), enabling us to analyze matrices that are larger than available physical memory. This required us to use SSD disk storage as it makes this analysis time-efficient as there is considerable disk IO associated with out-of-memory computation. Each biglasso model was fit for a series of values of the regularization parameter, $\lambda$. In the model selection dataset, we identified the optimal value of the regularization parameter to minimize root mean square error for each of the 6 SNP sets, and we further identified which p-value threshold gave the best results (**Table C in S1 Tables**). The regularization parameter controls the number of SNPs contributing to the prediction models, and this varied from 40,864 to 6,823 across the 6 SNP sets.

### Traditional Polygenic risk score models

Polygenic risk scores were built by calculating weighted sums of the number of SOS-reducing alleles (0, 1 or 2 for each SNP) carried by each person. The effect estimates from the SOS GWAS in this study was used as weights [6]. Six different p-value thresholds were used (**Table C in S1 Tables**), and then these SNP sets were filtered to eliminate highly correlated SNPs using PLINK LD clumping. For example, a p-value threshold of 5e-3, 44,091 SNPs were used to build the polygenic risk score; in contrast for a p-value threshold of 5e-8, only 1893 SNPs were used.

## FRAX Clinical Risk Factors in UK Biobank

To identify smokers in the cohort we use UK Biobank data fields 1239 (“Current Tobacco Smoking”, recording smokers as those answering “Yes, on most or all days”) and 20116 (“Smoking status”, recoding smokers as those marked “Current”). Self-reported rheumatoid arthritis was not recorded, as this is often confused with osteoarthritis [7].

### Glucocorticoid use

Each individual has a list of “Treatment/medication” codes of length greater than or equal to zero. Individuals having at least one of the following codes in their list was assigned a positive flag for glucocorticoid use: '1140868364', '1140874930', '1140874950', '1140874954', '1140888628', '1140874956', '1140874976', '1140883026', '1141157402', '1140874896', '1140884704', '1140910424', '1140910484', '1141157294' and '1141173346'. These codes represent medications ‘prednisone’, ‘prednisolone’, ‘prednesol 5mg tablet’, ‘hydrocortistab 20mg tablet’, ‘hydrocortistab 1% cream’, ‘hydrocortone 10mg tablet’, ‘methylprednisolone’, ‘methylprednisolone+neomycin’, ‘prednisolone product’, ‘hydrocortisone’, ‘cortisone product’, ‘hc - hydrocortisone’, ‘cortisol product’, ‘hydrocortisone product’ and ‘cortisone’ respectively.

### Definition of Rheumatoid Arthritis

ICD10

M06 = Other rheumatoid arthritis

M06.0 = Seronegative rheumatoid arthritis

M06.00 = Seronegative rheumatoid arthritis (multiple sites)

M06.01 = M06.01 Seronegative rheumatoid arthritis (Shoulder region)

M06.02 = Seronegative rheumatoid arthritis (Upper arm)

M06.03 = Seronegative rheumatoid arthritis (Forearm)

M06.04 = Seronegative rheumatoid arthritis (Hand)

M06.05 = Seronegative rheumatoid arthritis (Pelvic region and thigh)

M06.06 = Seronegative rheumatoid arthritis (Lower leg)

M06.07 = Seronegative rheumatoid arthritis (Ankle and foot)

M06.08 = Seronegative rheumatoid arthritis (Other)

M06.09 = Seronegative rheumatoid arthritis (Site unspecified)

M06.8 = Other specified rheumatoid arthritis

M06.80 = Other specified rheumatoid arthritis (Multiple sites)

M06.81 = Other specified rheumatoid arthritis (Shoulder region)

M06.82 = Other specified rheumatoid arthritis (Upper arm)

M06.83 = Other specified rheumatoid arthritis (Forearm)

M06.84 = Other specified rheumatoid arthritis (Hand)

M06.85 = Other specified rheumatoid arthritis (Pelvic region and thigh)

M06.86 = Other specified rheumatoid arthritis (Lower leg)

M06.87 = Other specified rheumatoid arthritis (Ankle and foot)

M06.88 = Other specified rheumatoid arthritis (Other)

M06.89 = Other specified rheumatoid arthritis (Site unspecified)

M06.9 = Rheumatoid arthritis, unspecified

M06.91 =  Rheumatoid arthritis, unspecified (Shoulder region)

M06.92 = Rheumatoid arthritis, unspecified (Upper arm)

M06.93 = Rheumatoid arthritis, unspecified (Forearm)

M06.94 = Rheumatoid arthritis, unspecified (Hand)

M06.95 = Rheumatoid arthritis, unspecified (Pelvic region and thigh)

M06.96 = Rheumatoid arthritis, unspecified (Lower leg)

M06.97 =  Rheumatoid arthritis, unspecified (Ankle and foot)

M06.98 = Rheumatoid arthritis, unspecified (Other)

M06.99 = Rheumatoid arthritis, unspecified (Site unspecified)

ICD9

714 = Rheumatoid arthritis and other inflammatory polyarthropathies

714.0 = 714.0 Rheumatoid arthritis

714.00 = Rheumatoid arthritis (multiple sites)

714.01 = Rheumatoid arthritis (shoulder region)

714.02 = Rheumatoid arthritis (upper arm)

714.03 = Rheumatoid arthritis (forearm)

714.04 = Rheumatoid arthritis (hand)

714.05 = Rheumatoid arthritis (pelvic region and thigh)

714.06 = Rheumatoid arthritis (lower leg)

714.07 = Rheumatoid arthritis (ankle and foot)

714.08 = Rheumatoid arthritis (other specified site)

714.09 = Rheumatoid arthritis (site unspecified)

714.2 = 714.2 Other r.a. with visceral or systemic involvement

714.21 = Other r.a. with visceral or systemic involvement (shoulder region)

714.22 = Other r.a. with visceral or systemic involvement (upper arm)

714.23 = Other rheumatoid arthritis with visceral or systemic involvement (forearm)

714.24 = Other rheumatoid arthritis with visceral or systemic involvement (hand)

714.25 = Other r.a. with visceral or systemic involvement (pelvic region and thigh)

714.26 = Other r.a. with visceral or systemic involvement (lower leg)

714.27 = Other r.a. with visceral or systemic involvement (ankle and foot)

714.28 = Other r.a. with visceral or systemic involvement (other specified site)

714.29 = Other r.a. with visceral or systemic involvement (unspecified site)

### Secondary Causes of Osteoporosis

Individuals reporting type 1 diabetes, menopause prior to age 45, chronic liver disease or osteogenesis imperfecta were recorded as having a secondary cause of osteoporosis.

### Type 1 diabetes

Type-1 diabetes was defined as follows: Individuals having a self-reported non-cancer illness code '1222' in data-field 20002 were assigned a positive type-1 diabetes status. Others were assigned a negative status. 

Menopause prior to age 45
Women who indicated that their periods had stopped through a touchscreen questionnaire (Data-Field 2724) were asked at what age this occurred (Data-Field 3581). Women who answered “Do not know” or “Prefer not to answer” were assigned a value of zero. Women who provided an age greater than or equal to 45 were assigned a value of 0. Women who provided an age less than 45 were assigned a value of 1. All other individuals in the cohort were assigned a value of zero. 

Chronic liver disease
Individuals having one of the following ICD9 codes were assigned a positive chronic liver disease status: 571, 5710, 5711, 5712, 5713, 5714, 5715, 57150, 57151, 57152, 57158, 57159, 5716, 5717, 5718, 5719. These ICD9 codes correspond to ‘Chronic liver disease and cirrhosis’, ‘Alcoholic fatty liver’, ‘Acute alcoholic hepatitis’, ‘Alcoholic cirrhosis of liver’, ‘Alcoholic liver damage, unspecified’, ‘Chronic hepatitis’, ‘Cirrhosis of liver without mention of alcohol’, ‘Cirrhosis of liver without mention of alcohol (congestive)’, ‘Cirrhosis of liver without mention of alcohol (postnecrotic)’, ‘Cirrhosis of liver without mention of alcohol (childhood function)’, ‘Portal fibrosis without cirrhosis of liver without mention of alcohol’, ‘Cirrhosis of liver without mention of alcohol (other and unspecified)’, ‘Biliary cirrhosis’, ‘Other chronic nonalcoholic liver disease’, ‘Unspecified chronic liver disease without mention of alcohol’, respectively. 

Osteogenesis imperfecta
Individuals having one or both of the ICD9 code 75650 and ICD10 code Q780 were assigned a positive osteogenesis imperfecta status. The codes correspond to ‘Osteodystrophies (osteogenesis imperfecta)’ and ‘Osteogenesis imperfecta’, respectively. All other individuals in the cohort were assigned a negative osteogenesis imperfecta status.

# References

1. Morris JA, Kemp JP, Youlten SE, Laurent L, Logan JG, Chai RC, et al. An atlas of genetic influences on osteoporosis in humans and mice. Nat Genet. 2018; 338863. doi:10.1038/s41588-018-0302-x

2. Abraham G, Inouye M. Fast principal component analysis of large-scale genome-wide data. PLoS One. 2014;9: 1–5. doi:10.1371/journal.pone.0093766

3. Loh P-R, Tucker G, Bulik-Sullivan BK, Vilhjálmsson BJ, Finucane HK, Salem RM, et al. Efficient Bayesian mixed-model analysis increases association power in large cohorts. Nat Genet. 2015;47: 284–290. doi:10.1038/ng.3190

4. Tibshirani R. Regression Selection and Shrinkage via the Lasso. Journal of the Royal Statistical Society B. 1996. pp. 267–288. doi:10.2307/2346178

5. Zeng Y, Breheny P. The biglasso Package: A Memory- and Computation-Efficient Solver for Lasso Model Fitting with Big Data in R. 2017 [cited 31 Oct 2019]. Available: http://arxiv.org/abs/1701.05936

6. Evans DM, Visscher PM, Wray NR. Harnessing the information contained within genome-wide association studies to improve individual prediction of complex disease risk. Hum Mol Genet. 2009;18: 3525–3531. doi:10.1093/hmg/ddp295

7. Kriegsman DMW, Penninx BWJH, Van Eijk JTM, Boeke AJP, Deeg DJH. Self-reports and general practitioner information on the presence of chronic diseases in community dwelling elderly: A study on the accuracy of patients’ self-reports and on determinants of inaccuracy. J Clin Epidemiol. 1996;49: 1407–1417. doi:10.1016/S0895-4356(96)00274-0
