# Supplementary material for: Development of a polygenic risk score to improve screening for fracture risk: A genetic risk prediction study
Source: PLoS Med. 2020 Jul 2;17(7):e1003152. doi: 10.1371/journal.pmed.1003152 (PMC7331983; doi:10.1371/journal.pmed.1003152)
Supplement: Attachment — Submitted filename: r1-PLOSMED-gSOS-rebuttal.docx [file pmed.1003152.s008.docx]

We thank the Editors and Reviewers for their helpful comments concerning our article. We have provided responses to these queries below, directly underneath the original comments. Our responses are in **blue bold font** to improve readability and all changes to the manuscript have been denoted by page and line number.
-------------------------------------------------------------------------------------------------------------------------------
Requests from the editors:

Throughout your text, please adapt the wording to reflect the experimental design rather than anticipations of possible use dependent on further clinical studies. For example, "The use of a polygenic risk score ... can reduce ..." seems too stridently worded.

Thank you for your comment, we have revised the manuscript to more clearly state the results obtained in this study. The following was edited (*original followed by the edit*):

*“…we found that the use of a polygenic risk score in fracture risk screening can decrease the…”*

“…we found that the use of a polygenic risk score in fracture risk screening decreased the…”

*“…we provide evidence in 5 separate cohorts…”*

“…we determined using 5 separate cohorts…”

*“…they suggest a role for polygenic risk scores…”*

“…they do demonstrate the utility of polygenic risk scores…”

We welcome any additional edits you propose.

Please add a study descriptor to the title. We suggest: "Development of a polygenic risk score to improve screening for fracture risk: a cross-sectional study".

**We would argue that no PRS study is cross-sectional. This is because the exposure (in this case genotypes) is present from birth and therefore not ascertained at the same time as the outcome (in this case BMD). Rather, the exposure does not change with time, which is of course one of the main advantages of genetic studies. Therefore, we would argue not to change the title to include the words “a cross-sectional study”. Should you require a study descriptor, we recommend “A cohort study with replication”.**

Please convert the abstract to PLOS Medicine style, i.e., with three subsections. The final sentence of the "methods and findings" subsection should summarize the study's main limitations.

The abstract has now been modified to the PLOS Medicine style.

Please begin the "Conclusions" subsection of your abstract with "In this study, we found that ..." or similar.

The Conclusions section of the Abstract now states, “In this study we found that the use of a polygenic risk score in fracture risk screening…”.

Please quote summary demographic and clinical details for study participants in your abstract.

We have now added the sentence:

“Across the five cohorts, the average age ranged from 57-75 years and 54% of studied individuals were women.”

In your abstract and elsewhere in the paper, please add p values alongside 95% CI where available.

The 95% CI’s provide more information than a p-value. A p-value only provides information on whether the null hypothesis can be accepted or rejected. Confidence intervals on the other hand provide information regarding the range of values within which the true value plausibly lies, the strength of the effect and whether the null hypothesis can be rejected. While we agree that p-values can be helpful in large-scale hypothesis testing, such as GWAS, the results presented in our study are not a GWAS, rather they are testing the effect of a polygenic risk score to improve screening for fracture. This is the reason why many journals prefer the reporting of confidence intervals[1]. Also, PLOS Medicine submission guidelines suggests the use of confidence intervals:

*“…When possible, quantify findings and present them with appropriate indicators of measurement error or uncertainty (such as confidence intervals). Avoid relying solely on statistical hypothesis testing, such as P values, which fail to convey important information about effect size and precision of estimates…”*

https://journals.plos.org/plosmedicine/s/submission-guidelines#loc-methods

After your abstract, we will need to ask you to add a new and accessible "author summary" section in non-identical prose. You may find it helpful to consult one or two recently published research papers in PLOS Medicine to get a sense of the preferred style. 

We have added an author summary to the manuscript following the Abstract.

Early in your methods section, please state whether the study had a protocol or prespecified analysis plan and if so attach the relevant document(s) as a supplementary file (referred to in the text). Please highlight analyses that were not prespecified. 

This study did not have a pre-specified analysis plan.

Please remove trademarks from the manuscript.

Trademarks removed.

Throughout the text, please format reference call-outs as follows: "... that for BMD [10,24]."

Callouts formatted as per PLOS Medicine style.

Please ensure that citations in your reference list meet journal style. All boldface and italic elements should be converted into plain text. Where appropriate, 6 author names should be listed, followed by "et al.".

Reference list formatted as per PLOS Medicine style.

Please add a journal name to reference 37 as appropriate. 

Reference updated with journal name and conformance to PLOS Medicine guidelines.

If references 41 and 43 are preprints, please indicate this. 

BioRxiv references are indicated as preprint.

Please add a completed checklist for the most appropriate reporting guideline, which may be STROBE, referred to in your methods section. In the checklist, individual items should be referred to by section (e.g., "Methods") and paragraph number rather than by page or line numbers, as the latter generally change in the event of publication. 

We did not use STROBE guidelines, as those are for observational studies. We chose to use GRIPS checklist as is it recommended for genetic risk prediction studies, and was published by PLOS Medicine [ref: <https://doi.org/10.1371/journal.pmed.1000420> ]

The GRIPS checklist is provided as a supplementary table that is referred to in the Methods section.

Comments from the reviewers:

*** Reviewer #1: 

The authors present a well-written and interesting study on developing a polygenic risk score for heal quantitative ultrasound speed of sound (SOS) for identify low risk individuals who can excluded from fracture risk screening. The strengths of the study include, developing and testing the genetic risk score (GRS) in a large population cohort (UK Biobank), interestingly using both a LASSO-based data-driven approach, and then validating the performance of the GRS in 5 other cohorts with FRAX-score risk factors available , including Bone Mineral Density. The key finding being that there were substantial reductions in proportion of individuals requiring FRAX-based tests (with or without BMD).
There are quite a few analyses in this and some of the results are difficult to follow as they are given in excel workbooks instead of word format. However, the statistical methods appear sound - with appropriate QC of genotyping data and SNP selection. The introduction of the LASSO model compared to simple polygenic risk score is also a nice addition and showed that using a data-driven approach can improve the variance in the model (up to 25%). 
Much of the literature on polygenic risk scores has been done in the context of "how much added values does GRS have above and beyond a clinical prediction model" with a focus on the overall change in AUC. The authors have framed their research question differently, in particular about how a GRS could preclude people from undertaking FRAX risk assessment (which have some level of costs). Whilst the modelling is convincing comparing these strategies - I am still left wondering about the clinical utility and feasibility of such an approach given that genotyping will need to be done on ALL individuals. Albeit the authors mentioned genotyping is more available now but that is really limited still to research based studies - it not a strategy which is indeed offered as routine clinical care. 
Specific comments:
(1) The project clearly shows the potential of using polygenic risk score but it would be useful if the authors could comment about the clinical utility of their approach. There is a growing drive for genomic testing in routine practice but given that their LASSO model included 21,717 activated SNPs mean that's genotyping arrays of this magnitude will need to be utilised - which will indeed a barrier for implementation. The authors themselves had to use Amazon cloud computing to conduct the analyses. I'm wondering if a reduced set of SNPs would confer similar set of results. In Table S2, the LASSO approach with 6,823 SNPS explains about 23.4% which is not far off the 24.99% achieved by having 21,717 SNPs. There is in fact an argument for using more simplistic GRS scores with fewer SNPS.

This is a relevant point and one that deserves discussion. We agree that more parsimonious models are better, in general. However, in this case, the improved prediction accuracy by using more SNPs provides justification for less parsimonious model. Below, we offer three reasons why this is the case.

First, the financial cost of assaying 21,717 versus 6,823 is the same. This is because both models would rely upon genome-wide genotyping, followed with imputation to the entire imputation panel.

Second, the computational cost is the same. No user of the gSOS model would require use of expensive high-performance computational servers, such as were used in our paper. This is because such servers were used only to generate the gSOS model. Now that the gSOS model has been generated, it can be computed on virtually any computational device, including laptops, or even smart phones.

Third, an investment made in genome-wide genotyping an individual could also be used to predict other outcomes, other than gSOS, thus further lowering the incremental cost of implementing genomically-informed clinical care.

Of minor note, we have edited the supplemental text to further elaborate the computational requirements of biglasso, as the use of “Amazon” is simply a vendor and does not specify the motivation and solution:

“The biglasso R package uses an out-of-memory matrix (implemented by the bigmemory R package), enabling us to analyze matrices that are larger than available physical memory. This required us to use SSD disk storage as it makes this analysis time-efficient as there is considerable disk IO associated with out-of-memory computation.”

**We have now clarified the above concerns by including the following sentences in the Discussion, on page 12, line 413:**

“We observed similar predictive performance across all LASSO models in the model selection step (Table S3 in current revision); therefore, it remains possible that a more parsimonious model containing fewer SNPs would perform almost as well. As a result, further exploration of the LASSO models we have developed is warranted in a future technical study. However, should a more complex model with more SNPs prove to be ideal, the hinderance to clinical translation will be minimal, as the computational burden is limited to the training of the models, and not in the prediction of an individual’s genetic risk.”

(2) Please check text results section (Lines 344) as it says the SNP array with 21,717 activated SNPS has a r^2 of 23.2% but in Table S2 the r^2 for the 21,717 SNPS

SNPs in each model (Table S3) are not on individual genotyping “arrays”. They are selected from the set of SNPs within the UK Biobank genotype imputation data set that achieve a minimum GWAS p-value for their association to SOS.

We assume the reviewer intended to state at the end of this comment that “the r^2 for the 21,717 SNPs … ” is 24.99% from Table S3.

The variance explained (VE) value of 24.99% is from the “Model Selection Set" (Table S3). The reported value in the main text of 23.2% is from the “Test Set”, which is also noted in Table S3.

(3) CRF-FRAX is significantly cheaper to apply without BMD - in fact many EHRs have this embedded within medical record. In the UK for instance, TPP offers CRF-FRAX and EMIS offers QFracture using data which already collected in patient's medical records. There is logical mismatch in argument made by the authors in the discussion on "requiring all individuals to undergo a CRF-FRAX test" as the scores are CRF-FRAX or QFractures scores are estimated instantaneously from the medical record. The reductions in individuals requiring CRF-FRAX is actually not a huge burden with algorithms being integrated into EHRs. The more compelling argument here is the reduction of BMD-based FRAX tests.

We agree that the more interesting findings for physicians practicing with an EHR that automatically generates CRF-FRAX results would be the reduction in BMD-based FRAX tests. However, the majority of practicing physicians in the world do not have access to an EHR that automatically generates CRF-FRAX results. Thus, the main discussion centers around a decrease in BMD-based FRAX tests. However there is no appreciable change in the sensivitiy and/or specificity to correctly identify individuals requiring therapy if the gSOS screening step was placed prior to the CRF-Frax test, or immediately after it.

Nevertheless, we accept that important proportion of the readership of this article would be physicians from the UK. Therefore, Supplementary Tables S6-S16 now display the results of reduction in BMD-based FRAX tests. Further, we have added the following sentence to the Results section on page 12, line 426:

“**We acknowledge that for many practicing physicians, having access to an automatically generated electronic health record-based CRF-FRAX test, the results of interest would be the reduction in the BMD-based FRAX tests. However, there was no appreciable difference in the sensitivity and/or specificity to correctly identify individuals requiring therapy if the gSOS screening step was placed prior to the CRF-FRAX test, or immediately after the CRF-FRAX test. Supplementary Tables S6-S16 show the results for a reduction in BMD-based FRAX tests by cohort and sex.”**

(4) I found it interesting the authors looked at placing the genomic screening step after the CRF-FRAX and similar to other studies which have looked at the added value of the GRS on top of the clinical prediction model, they found no further improvement in sensitivity and specificity. As a population screening model the key comparison should be the CRF-FRAX model as this has been embedded in primary care EHRs at very minimal costs. The current comparison is BMD-FRAX but the more commonly and cheaper alternate which is adopted in primary care is CRF-FRAX due to BMD not being routinely available.

We would like to clarify that when we stated that gSOS did not change the sensitivity and specificity, this was not for prediction of fracture. Rather, there was no change in the sensitivity and specificity to identify individuals who would be recommended treatment, when comparing whether gSOS was used before or after the CRF-FRAX step. Thus, for practical purposes the results do not change with respect to sensitivity and specificity to identify correct treatment allocation.

***** Reviewer #2:** 

Forgetta et al., 2019

Forgetta et al. analyze data from UK Biobank to derive a polygenic risk score (gSOS) for heel ultrasound speed of sound. They show that the polygenic risk score can be used to reduce the number of patients that require DXA scans for bone mineral density in the assessment of fracture risk. 

This paper tests gSOS in clinical cohorts. The authors show that gSOS can be used to eliminate the need for DXA scans for some patients, thereby decreasing the cost of screening a general population. The authors set a threshold for gSOS such that patients above the threshold are "reassured" and do not require further screening. The key result can be seen in Fig. 6, which shows that incorporating gSOS retains sensitivity and specificity but decreases the number of patients that require CRF-FRAX and BMD-FRX. The figure clearly shows the cost savings of genetic testing in diagnosing osteoporosis. 

I reviewed a previous version of this paper, and the current submission has already responded to many of my comments. 

Minor comments

Line 441 "Recently, LASSO regression was used to predict estimated BMD, but from a GWAS sample size that was one third of that used here, captured only 20% of the its variance and was not used in a screening program.41" This statement should refer to reference 14, which developed gSOS previously based on the identical UK Biobank cohort and with nearly identical performance (i.e. correlation to SOS) as in this submitted work.

We thank the reviewer for noting this. The citation is corrected to Kim et al. [14]. Also, the variance explained value from Kim et al. of 20% includes additional covariates, such as age, sex, height and weight (see BOG score in Kim et al. [14]). We have corrected this to report the variance explained by polygenic score only (r=0.415, r^2=0.172). Also, note that Kim et al. used eBMD which is a linear combination of BUA and SOS.

Given the above correction, the performance from Kim et al. is more similar to the PRSs that use SNP clumping (Table S3), and is substantially lower than our LASSO model for SNPs at P < 5e-4 (r=0.481 [0.467-0.487], Table S3).

In Table 1, some of the demographics differ between groups and should have comments:

One of the main reasons that we have included five separate cohorts is to test the generalizability of gSOS across these cohorts, which have different demographic characteristics. This allows physicians to assess the expected performance of gSOS in different patient populations.

1. The percent smoker in the Biobank test set is much different than the training or selection set. Smoker is a key factor for FRAX, and this difference could impact performance of gSOS. 

Yes, this is correct that the prevalence of smoking is increased in UK Biobank Test set, compared to the other cohorts and the UK Biobank Training and Model Selection sets. It is important recall that the UK Biobank Test set was not used to derive gSOS, but rather to test it. Therefore, systematic differences in the UK Biobank Test set, compared to training and model selection will not influence the generation of gSOS.

Nevertheless, Tables S6-S16 show the performance of gSOS is relatively stable across cohorts. For example, comparing the UK Biobank Test set to the overall estimates, shows that incorporating gSOS as a screening step rendered a sensitivity of 95.0% and specificity of 100% to identify individuals requiring treatment (Table S6). These estimates were similar when incorporating all replication cohorts, where the sensitivity was 93.4% and specificity 98.5% (Table S5). Therefore, while there is a difference in smoking status between the UK Biobank Test set and the other cohorts, this did not render major differences in the sensitivity and specificity of the gSOS screening step. This is of course likely because the UK Biobank Test set was not used to generate gSOS.

2. Age in the four clinical cohorts is much higher than the Biobank study.

This is correct. Indeed, we have specifically sought replication cohorts of increased age, because the UK Biobank has an age distribution which is lower than most osteoporosis clinics. The fact that gSOS replicates across cohorts emphasizes the generalizability of the findings to an age distribution more consistent with individuals who are seen in osteoporosis clinics.

3. The percent of previous fracture in the four clinical cohorts is higher than in the UK Biobank. Previous fracture is a very strong risk factor for osteoporotic fractures. This difference likely impacts the results. 

Again, Reviewer 2 is correct. Fracture risk is strongly associated with age, which is addressed in the comment below. This difference in fracture rates further increases the generalizability of our findings, allowing physicians to judge whether the results are applicable to their clinical population.

4. The percent of falls in the Biobank test set was higher than in the training or marker selection set. This is very worrisome as it suggests that the test set was at higher risk for fragility fracture than the training set.

Recall that the UK Biobank Test set was not used to generate gSOS. Therefore, differences in the percentage of falls in the UK Biobank Test set will not influence the performance of gSOS in other non-UK Biobank cohorts. The non-UK Biobank cohorts provide a range of prevalence of falls, from 10% in CLSA to 28% in SOF, allowing the reader to assess the performance of gSOS in different clinical populations.

Table 1 should list 95% CI for each of the percentages to show which of the difference are statistically significant.

“Statistical significance” testing would allow us to test whether there are underlying differences in risk factors in the populations from which the cohorts were sampled. The purpose of our work is not to test whether the risk factors in different populations are different. For similar reasons, the difference in risk factors between the placebo and intervention groups in randomized controlled trials are not presented in such studies.

Rather, the purpose of our replication testing across five separate cohorts, is designed to allow testing of the performance of gSOS across individuals of varying risk factors. The fact that the replication cohorts have different risk factor profiles allows readers to assess the generalizability our study, by enabling the reader to judge the performance across these different risk factor profiles. The reader can then pick a cohort that most resembles their clinical practice.

***** Reviewer #3:** 

Forgetta et al. have developed and tested a polygenic prediction tool for bone fracture, based on the large UK Biobank sample (341,449 individuals) and a polygenic risk score for heel quantitative ultrasound speed of sound (SOS). The paper is timely and interesting, and shows that genetic testing may reduce the need for additional screening tests. Some issues should be addressed:

1) The use case can be improved. The risk for an event is less clinically relevant than prediction the time of the event. It would be of interest to see the results with for example the polygenic hazard score in this context (published in PLOS Medicine in 2017). 

Thank you for this comment. We agree that time to event is more clinically relevant than risk of an event.

It would appear from Reviewer #3’s comment above, “Forgetta et al. have developed and tested a polygenic prediction tool for bone fracture…” that Reviewer #3 has understood that we are attempting to predict fracture. However, this is not the case. We have generated a predictor for a continuous trait: Speed of Sound (SOS) from heel ultrasound. Since this predictor is derived from genotypes alone, we have called this “gSOS”.

We have not used gSOS to predict fracture. Rather, we have used gSOS to understand if it can help to make a fracture risk screening program more efficient. Therefore, we cannot generate the “time to event”, because we are not predicting an “event”.

2) Further, in what age groups are the gSOS most predictive? This should be added to the model.

gSOS is not being used for the prediction of fracture, please see response to Comment #1. Related to this, gSOS is not correlated with age (see reply to Comment #7 below); however, age-stratified analysis of the CLSA cohort did reveal some difference in screening performance, across age, which is now reported in the manuscript (see reply to comment #4 and #6).

3) What is the positive predictive value of gSOS? These result should be presented.

We agree that positive predictive value would be useful if we were predicting risk of fracture. However, we are not predicting fracture risk, rather we are predicting a continuous trait, SOS, for which a positive predictive value is not helpful. Please see response to Comment #1.

4) Outcome: the sensitivity and specificity did not improve, while the proportion of individuals requiring CRF-FRAX testing decreased from 81% to 51% and the proportion of screened individuals requiring BMD-FRAX testing decreased from 37% to 22%. However, this seems to be age dependent, and for which age group is this relevant?

**This is an excellent question, which we did not explore. To answer this question we have undertaken stratified analyses in the CLSA cohort to understand the effect of age on the outcomes of our study: 1) the sensitivity and specificity to identify individuals who should be recommended to interventions and 2) the percent decrease in CRF-FRAX and BMD-FRAX testing due to the gSOS screening step. We chose the CLSA cohort because it is the largest cohort (N = 6,704) and has the largest age standard deviation (9.9 years).**

**Undertaking this stratified analysis demonstrated that the sensitivity and specificity to correctly identify individuals requiring an intervention was largest in individuals ≥70 years (see Table S7). This is clinically relevant since the best osteoporosis case-finding evidence we have is arguably from the SCOOP trial, which assessed individuals in this age-range. Therefore, gSOS may be particularly relevant in the individuals for whom we have randomized controlled trial evidence to support case-finding. We have now changed the text to state, on page 10, line 348 in the Results:**

**“We next tested the performance of gSOS by different age strata to understand if the screening efficiency improved more for one age group, rather than another. Using the largest cohort, with the largest variation in age (CLSA, N = 6,704), we found that individuals ≥70 years had the highest performance. Specifically, the sensitivity and specificity to identify individuals who require an intervention remained high at 99.6% and 94.9%, respectively. The proportion of screened individuals requiring CRF-FRAX testing decreased from 73% to 37% (representing relative decrease of 49%), when compared to NOGG guidelines without a gSOS screening step. Additionally, the proportion of screened individuals requiring BMD-FRAX testing decreased from 24% to 12% (representing a relative decrease of 50%) (Table S7). In contrast, individuals aged 50-59 years had a sensitivity reduced to 86%, but a specificity of 99.6%. The percent of individuals requiring CRF-FRAX and BMD-FRAX testing reduced by 51% and 50%, respectively. This demonstrates that gSOS pre-screening improves the efficiency of screening, but that the sensitivity to correctly identify individuals requiring therapy is maximized in older age groups. ”**

**We have also included the following in the Discussion, page 12, line 421:**

**“The sensitivity and specificity to correctly assign interventions was maximized in individuals ≥70 years of age. This could be clinically relevant because this is the age-range for which the SCOOP trial demonstrated that a community-based screening program could effective in reducing hip fractures.”**

5) The sensitivity and specificity of gSOS is not better than the for the other measures (a small decrease in sensitivity to 93.4%, while the specificity increased slightly to 98.5%). This result should be discussed, and the reason for lack of improvement should be presented.

The objective of this work is to estimate the number of clinical and BMD tests that can be avoided by incorporating a gSOS screening step, without suffering a large decrease in the sensitivity to identify individuals who should be treated. Again, we are not trying to predict fracture.

Therefore, the decrease in sensitivity when using a gSOS screening step reflects the proportion of the population who should have been assigned treatment, when in actual fact, they were assigned reassurance. Figure 6 demonstrates that, when compared to the usual NOGG guidelines, the sensitivity decreases marginally, but the specificity increases marginally. This is discussed in the first paragraph of the Discussion and the Conclusions of the Discussion.

6) Earlier intervention is often better for prevention. Does the gSOS improve the predictive value of BMD-FRAX or CRF-FRAX if applied to younger age groups? This is a highly relevant analysis.

**As now outlined in our response to comment #4 above, gSOS had different performance characteristics when stratified by age. However, again, we did not assess positive predictive value because we are not assessing fracture risk.**

7) UK Biobank - the GWAS did not include age as variable? Is it possible to provide result of age dependent GWAS, at least for different age groups?

The GWAS *did* include age, sex, genotype array, and UK Biobank assessment centre as covariates. Please refer to Methods in the main text (see page 7, line 171). Moreover, gSOS is not correlated to age as genetic determinants of SOS (gSOS) are present from birth and do not associate with age (Pearson r^2 = 0.0057, p-value = 0.106, plot below).

8) There should be non-European samples available that the authors can use for testing the performance in other ancestries.

**Thank you for this excellent point. We have now run the analyses on individuals of non-White British descent within the UK Biobank. As outlined in our manuscript, none of these individuals were included in the training or model selection sets, so represent an independent validation of the effect of gSOS pre-screening on the NOGG guidelines. These results are now in Supplementary Table 17 and demonstrate general consistency with the results from individuals of White British ancestry within the UK Biobank test set.**

**Specifically, we now include in the manuscript the following in the manuscript’s Results section:**

**“We then assessed the effect of a gSOS pre-screening on individuals from UK Biobank with DXA BMD measures who were of non-White British ancestry. We found that the results were generally consistent with individuals of White British ancestry. Specifically, the proportion of screened individuals requiring CRF-FRAX testing decreased from 94% to 48% (representing relative decrease of 49%), when compared to NOGG guidelines without a gSOS screening step. Additionally, the proportion of screened individuals requiring BMD-FRAX testing decreased from 39% to 17% (representing a relative decrease of 57%) (Table S17).**

**The proportion of CRF-FRAX and/or BMD-FRAX tests that resulted in an individual being excluded from the screening program without a recommendation for an intervention also decreased from 92% to 47% and from 38% to 16%, respectively. (Table S17).”**

**We agree that further work should be done looking at separate ancestries in larger populations and have added this to the limitations paragraph.**

**“While we have tested the utility of gSOS in individuals of non-White British ancestry, the sample size available for study was relatively small, and thus results should be replicated in additional cohorts of different ancestry, underlining the need for large-scale GWAS datasets in individuals of non-European ancestry”**

***

Any attachments provided with reviews can be seen via the following link:
********

__________________________________________________
In compliance with data protection regulations, you may request that we remove your personal registration details at any time. (Use the following URL: https://www.editorialmanager.com/pmedicine/login.asp?a=r). Please contact the publication office if you have any questions.

**References**

1.	Ranstam J. Why the P-value culture is bad and confidence intervals a better alternative. Osteoarthritis and Cartilage. 2012. doi:10.1016/j.joca.2012.04.001
