# Supplementary material for: Development of a polygenic risk score to improve screening for fracture risk: A genetic risk prediction study
Source: PLoS Med. 2020 Jul 2;17(7):e1003152. doi: 10.1371/journal.pmed.1003152 (PMC7331983; doi:10.1371/journal.pmed.1003152)
Supplement: Attachment — Submitted filename: r2-PLOSMED-gSOS-rebuttal.docx [file pmed.1003152.s009.docx]

**General editorial requirements**

In addition, we request that you upload any figures associated with your paper as individual TIF or EPS files with 300dpi resolution at resubmission; please read our figure guidelines for more information on our requirements: <http://journals.plos.org/plosmedicine/s/figures>.

While revising your submission, please upload your figure files to the PACE digital diagnostic tool, <https://pace.apexcovantage.com/> PACE helps ensure that figures meet PLOS requirements. To use PACE, you must first register as a user. Then, login and navigate to the UPLOAD tab, where you will find detailed instructions on how to use the tool. If you encounter any issues or have any questions when using PACE, please email us at [PLOSMedicine@plos.org](mailto:PLOSMedicine@plos.org).

**Images have been converted to TIF using PACE and included as part of this submission.**

**Requests from the editors**

To your data statement, please add the UK Biobank contact details for those wishing to inquire about access to data.

**We had added this to the data statement within the manuscript portal.**

Please add a study descriptor to your title, following a colon, e.g. "...: a genetic risk prediction study".

**We have changed the title to include this descriptor.**

Please add a new concluding sentence to the "methods and findings" subsection of your abstract, quoting 2-3 of the study's main limitations.

**We have added two sentences as follows:**

***“This polygenic risk score could be improved by testing its performance in individuals of non-European ancestry. It’s clinical utility could also be improved by creating a polygenic scores based on DXA-BMD.”***

If not done, please add a sentence to the methods section to note that the study did not have a pre-specified analysis plan.

The following was added to the methods section:

**“… and did not have a pre-specified analysis plan.”**

Throughout the paper, please format reference call-outs as follows, preceding punctuation: "... [1].".

**Manuscript call-outs have been formatted as requested.**

Please add p values alongside 95% CI, where available.

**When computing p-values for the Pearson correlation coefficients we noted that all values are well below 10^-100^. Due to large size of the test set (N=80,027) any substantial correlation will be highly significant. The first observable p-value > 0 is at a r^2^ value of 0.01, which is below any correlation that this study reports. The following R code demonstrates this:**

```
> r <- sqrt(0.01)
> n <- 80027
> z <- 0.5 * log((1+r)/(1-r))
> zse <- 1/sqrt(n-3)
> min(pnorm(z, sd=zse), pnorm(z, lower.tail=F, sd=zse))*2
      [1] 3.243466e-177
```

**Given the above we request that p-values be omitted for these results, as they become meaningless at numbers so close to zero.**

Please remove all trademarks, e.g., at line 67.

**The single instance of a trademark was removed.**

Please present the reporting checklist in a separate supplementary document, referred to in your methods section.

**The GRIPS checklist was migrated to the Supplement.**

Comments from the reviewers:

Reviewer #3

The authors have addressed my comments adequately, except for the points about prediction which seems to be based on a misunderstanding.

I understand they are not predicting fracture. However, they are predicting an event: who requires an intervention ("be eligible for an intervention", "identify individuals requiring treatment".)

They are estimating the sensitivity and specificity for this event.

Thus, they should be able to provide

1) Estimate the time to the event (requiring treatment), or this is a limitation

2)	The age group where the sensitivity and specificity is highest for the event (to identify individuals requiring treatment),

3)	The positive predictive value for the event (requiring treatment)

They should provide standard clinical measures for prediction performance to identify individuals who require an intervention based on their quantitative measure. Otherwise, the approach has no clinical interest

**We would like to thank Reviewer #3 for their continued interest in improving our study. We have now added the positive predictive value for requiring treatment, as suggested to Supplementary Tables S4-S16, thereby providing this metric for all cohorts and men and women separately.**

**The following text has now been added to the paper:**

**“The positive predictive value for correct treatment assignment in all validation cohorts was 91.8% without a gSOS screening step and increased to 95.4% with the gSOS screening step (Table S4 cohort-level results and subgroup results are available in Tables S4-S16).” Page 12, line 329.**

**The age group where the sensitivity and specificity is highest for the event is demonstrated in Supplementary Table S6. The age group is individuals ≥70 years, which is particularly relevant for screening, since this is the age group for which there is best evidence to implement screening. This is discussed in a full section devoted to “Stratification by Age” in the results section.**

**The first point brought up by Reviewer #3 concerns the estimated time to event, where the event is the screening procedure to identify who requires treatment. This cannot be predicted from genetic variables, since the age at which the screening procedure is implemented is determined by National Osteoporosis societies through their guidelines.**
